# Aggressive Palliation in Extensive Stage Small Cell Lung Cancer, Practice Guidelines versus Clinical Practice: A Case Report and Review of the Literature

**DOI:** 10.4061/2011/659807

**Published:** 2011-05-15

**Authors:** Edward F. Miles, Laura L. Jacimore, John W. Nelson

**Affiliations:** ^1^Division of Radiation Oncology, Department of Radiology, Naval Medical Center Portsmouth, 620 John Paul Jones Circle, Portsmouth, VA 23708, USA; ^2^Division of Radiation Oncology, Nash General Hospital, 2460 Curtis Ellis Drive, Rocky Mount, NC 27804, USA

## Abstract

Small cell lung cancer (SCLC) constitutes approximately 16% of all primary lung cancers, with more than 35,000 new cases per year. Two-thirds of patients present with extensive stage disease (ES-SCLC) due to a tendency to metastasize early. Outcomes remain poor, with a median survival of approximately 10 months and a two-year overall survival of <10%. Current recommendations call for combination chemotherapy alone in patients without localized symptoms. Thoracic radiation therapy following a good clinical response is controversial. We report on a patient with ES-SCLC that had an excellent response to chemotherapy and underwent whole brain radiotherapy for a known brain metastasis and consolidative radiotherapy to the thorax. His latest follow-up demonstrates only a stable residual pulmonary nodule and no evidence of active metastatic disease. ES-SCLC is a relatively common presentation with a variable burden of metastatic disease. In the absence of randomized trials demonstrating the efficacy of thoracic radiation therapy, the community radiation oncologist is placed in a difficult position when addressing these patients, particularly those with otherwise good performance status and a good response to initial systemic chemotherapy. More research in this area is sorely needed to help guide treatment recommendations.

## 1. Introduction

Small cell lung cancer (SCLC) constitutes approximately 16% of all primary lung cancers, accounting for more than 35,000 cases per year [1]. Two-thirds of patients present with extensive stage disease (ES-SCLC) due to a tendency to metastasize early to distant sites, including the brain, bones, liver, and adrenals [2]. Outcome in ES-SCLC remains poor, with a median survival of approximately 10 months and a two-year overall survival of <10% [3]. Management has evolved over the past 30 years but has done little to improve clinical outcome. Current practice guidelines in ES-SCLC recommend combination chemotherapy alone [2, 4] in patients without localized symptoms (such as superior vena cava syndrome, lobar obstruction, or painful bone metastases) or chemotherapy with or without whole brain radiation therapy in the case of brain metastases. Delivering thoracic radiation therapy following a good clinical response to systemic chemotherapy is controversial.

Herein, we report a case of a patient diagnosed with ES-SCLC in a community hospital with extensive pulmonary disease, mediastinal involvement, an adrenal metastasis, and a single brain metastasis. 

## 2. Case Report

The patient was a 67-year-old African-American gentleman who presented to his local community hospital emergency department complaining of an abrupt onset of right arm weakness and dizziness which resulted in a fall with associated head injury. The patient denied specific loss of consciousness or seizure activity. A chest X-ray at his initial evaluation demonstrated a nodule in the left upper lobe. A computed tomography (CT) scan of the brain showed a single left frontal lobe mass with vasogenic edema. CT scans of the chest, abdomen and pelvis revealed mediastinal adenopathy, and two lesions in the left upper lobe, consistent with a primary lung disease. A follow-on MRI of the brain demonstrated a single 2.1 cm enhancing lesion in the left precentral gyrus. A CT-guided fine-needle aspirate of the lung lesion demonstrated small cell lung cancer. 

He met with a medical oncologist who outlined a treatment plan consisting of systemic chemotherapy followed by radiation to the brain and possibly concurrent radiochemotherapy to the chest. Immediately following his first round of systemic chemotherapy, a staging PET/CT demonstrated two hypermetabolic pulmonary lesions, the largest measuring 3.5 cm, extensive bilateral mediastinal and hilar activity, and a PET-avid right adrenal mass. He completed three cycles of a “programmatic”, locally defined cisplatin and irinotecan regimen with near complete resolution of his right-sided arm weakness. Restaging brain MRI demonstrated interval shrinkage in his single lesion and no further intracranial progression. A chest CT demonstrated a reduction in size of the lung lesions and resolution of the hilar and mediastinal adenopathy. 

Prior to his next planned cycle of chemotherapy, he was seen in the Radiation Oncology Department. His complete history was reviewed, and he was staged with ES-SCLC or Stage IV, T3N2M1 by AJCC criteria [2]. After a discussion of the risks and benefits, the patient was treated to a C1-whole brain field using opposed lateral fields, angled posteriorly to avoid divergence anteriorly into the orbits using 250 cGy fractions to a total dose of 3,500 cGy. He had an excellent response to his cranial irradiation with subsequent MRI showing complete resolution of the known lesion and no new progression. 

His PET/CT scan after completion of three cycles of chemotherapy showed resolution of the contralateral hilar disease as well as the subcarinal and adrenal disease. Due to his excellent response and good overall functional status, he was offered thoracic radiation, limited to the remaining PET-avid areas, all of which were confined to his left thorax. His planning CT scan was merged with his postchemotherapy PET/CT images, and the PET-avid areas were contoured as the gross tumor volume (GTV). A 1.5 cm margin was added for clinical target volume (CTV) with an additional 0.5 cm margin to arrive at a planning target volume (PTV). He was treated with an APPA field arrangement using 180 cGy fractions to 6,120 cGy concurrently with three addition cycles of cisplatin and irinotecan. Imaging studies completed near the end of his thoracic irradiation showed no evidence of further disease progression. He continues to do well clinically although he does continue to smoke. His latest imaging studies, a CT of the chest and an MRI of the brain, occurred approximately seven months after completing radiation therapy and showed a stable pulmonary nodule and no evidence of intracranial progression. His current followup regimen is a physical exam and chest CT scan every two months with a brain MRI every other visit. 

## 3. Discussion

Optimal treatment of patients diagnosed with ES-SCLC is controversial. There have been multiple, well-conducted studies over the past 40 years attempting to identify effective systemic and local combined therapies for this disease, yet both the management and ultimate outcome remain essentially unchanged over the interval [3]. The only exception to this has been a significant survival advantage in patients receiving prophylactic cranial irradiation following a good initial response to chemotherapy and negative brain imaging at the time of treatment [5] (unlike the patient presented herein). Radiation therapy has historically been reserved for palliation in patients with brain metastases or other sites of symptomatic metastases or primary bulky disease. However, asymptomatic patients are being increasingly offered thoracic radiation therapy based on a complete response (CR) or near CR to initial chemotherapy.

After tissue diagnosis was confirmed, this patient was appropriately staged with a complete history and physical exam, a complete set of laboratory studies, a dedicated thoracic CT scan with attention to the adrenal glands, a PET/CT scan, and a brain MRI. Unfortunately, as is all too common with this disease, his workup revealed multiple sites of metastatic disease. Although this patient clearly had extensive stage disease, due to the limited burden and small number of disease sites, he could be considered to be on the oligometastatic side of the extensive stage spectrum. In an ideal setting, prior to the initiation of any therapy, the management approach to this patient would have been formulated in a multidisciplinary setting with representatives from Medical Oncology, Radiation Oncology, Neuroradiology, and Pathology Departments. However, such an approach is not always available in the community hospital setting. Per the NCCN and ACCP guidelines, the standard of care for first line treatment of ES-SCLC is platinum-based systemic chemotherapy [2, 4]. The NCCN guidelines indicate that for patients with localizing symptoms (superior vena cava syndrome, lobar obstruction, or painful bone metastases), concurrent radiation therapy can also be considered. Radiation therapy upfront can be considered for spinal cord compression. In the setting of known brain metastases, historically present in approximately 10% of ES-SCLC patients at the initial presentation [6], whole brain radiation therapy is generally offered for symptomatic lesions prior to systemic chemotherapy. However, radiation therapy can be delayed until after systemic therapy for asymptomatic brain metastases. While this patient was initially symptomatic from his single brain metastasis (upper extremity weakness and dizziness), his symptoms responded well to high-dose oral corticosteroids. This, coupled with his wide-spread systemic disease, prompted the decision by his medical oncologist to initiate platinum-based systemic chemotherapy prior to initiation of radiation therapy.

Restaging studies, after completing three rounds of chemotherapy, showed a complete response in the adrenal gland, and partial response in the brain and thorax, at which time Radiation Oncology Department was consulted. As discussed above, he was treated to a standard whole brain field which he tolerated well. His performance status at this point was excellent, and both the patient and his medical oncologist strongly desired to consider thoracic radiation therapy concurrent with his ongoing chemotherapy. CT-based planning was used in the development of the final thoracic treatment fields. Initial attempts to include the original PET-avid extent of disease in the radiation fields required treating the contralateral hilum and mediastinum, resulting in a prohibitively high risk of radiation pneumonitis. We next merged his restaging PET/CT with the planning CT and defined GTV as all remaining PET-avid disease, with margin added as described above to arrive at a PTV. This approach limited his composite lung volume receiving 20 Gy or more (V20) to 6%, with an ipsilateral lung V20 of 14%; based on these values, we estimated his risk of symptomatic radiation pneumonitis to be acceptably low [7]. 

In selected patients with low-bulk disease and a complete, or near complete response after systemic chemotherapy, sequential thoracic chemoradiation therapy is a reasonable option and is supported by Phase III data. As reported by Jeremic et al., ninety-nine patients with ES-SCLC (albeit without brain metastases) who achieved a complete response outside the chest and at least a partial response in the chest to three cycles of systemic chemotherapy (carboplatin and etoposide) were randomized to receive thoracic chemoradiotherapy (5,400 cGy delivered in 150 cGy fractions twice daily with two concurrent cycles of chemotherapy) or four additional cycles of chemotherapy alone. They found a statistically significant improvement in median survival (17 months versus 11 months *P* = .041), median time to local recurrence (30 versus 22 months, *P* = .062), and five-year overall survival rate (9.1% versus 3.7%, *P* = .041) with thoracic chemoradiotherapy [8]. However, it must be noted that in the 50% of patients (initial prechemotherapy cohort was 210 patients) that had not achieved the necessary randomization criteria, there was little to no demonstrated benefit associated with the addition of thoracic radiation therapy and that four other randomized trials reported in the literature addressing this issue were negative studies [9].

Kochhar and colleagues published the Mayo clinic's experience with ES-SCLC with brain only metastases at initial diagnosis [6]. Of 30 patients evaluated, 16 had a single brain lesion; 19 received concurrent thoracic radiochemotherapy after 3 cycles of chemotherapy alone (delivered as part of a local protocol). All patients received WBRT (16–18 Gy) concurrently with initial chemotherapy cycles. Patients who received thoracic radiation tended to have a longer median survival than those that did not (16 versus 12 months). Median survival of patients with a single brain lesion was 14 months, equal to that of patients with limited stage SCLC. Given the generally poor outcome of patients treated with systemic chemotherapy alone, we felt it reasonable in this patient with otherwise asymptomatic disease and good response to chemotherapy outside the thorax to offer consolidative radiation therapy to the thorax. 

There is an on-going Phase II randomized trial (RTOG 0937) designed to evaluate the addition of thoracic radiation therapy in patients with ES-SCLC. It requires a radiographic partial or complete response to upfront chemotherapy with no evidence of progression. While multiple sites of metastatic disease are allowed, this study excludes patients with brain metastases. The primary endpoint is overall survival and is expected to accrue in 154 patients. A similar Phase II trial is underway in Canada (Alberta Health Services, LU-23929) but with an expected enrollment of only 30 patients and an endpoint of local control.

In conclusion, ES-SCLC is a relatively common presentation with a variable burden and distribution of metastatic disease. Current treatment recommendations result in relatively poor outcomes for the vast majority of patients. As the efficacy of systemic therapy improves with new agents and new combinations of agents, the value of local control using consolidative radiation therapy should also increase. In the absence of large randomized trials documenting the efficacy of consolidative thoracic radiation therapy, particularly in those patients that present with brain metastases, the community hospital radiation oncologist is placed in a difficult position when addressing these patients, particularly those with otherwise good performance status and a good response to initial systemic chemotherapy. As recently noted by Jeremic et al. [10], more research in this area is sorely needed to help guide treatment recommendations.

##  Disclaimer

The views expressed in this paper are those of the author(s) and do not necessarily reflect the official policy or position of the Department of the Navy, Department of Defense, or the United States Government. I am a military service member. This work was prepared as part of my official duties. Title 17 U.S.C. 105 provides that “Copyright protection under this title is not available for any work of the United States Government.” Title 17 U.S.C. 101 defines a United States Government work as a work prepared by a military service member or employee of the United States Government as part of that person's official duties.

##  Conflict of Interests

The authors declare that there is no conflict of interests.
